# Analysis of 7,815 cancer exomes reveals associations between mutational processes and somatic driver mutations

**DOI:** 10.1371/journal.pgen.1007779

**Published:** 2018-11-09

**Authors:** Rebecca C. Poulos, Yuen T. Wong, Regina Ryan, Herbert Pang, Jason W. H. Wong

**Affiliations:** 1 Prince of Wales Clinical School and Lowy Cancer Research Centre, Faculty of Medicine, UNSW Sydney, NSW, Australia; 2 Children's Medical Research Institute, Faculty of Medicine and Health, The University of Sydney, NSW, Australia; 3 School of Public Health, Li Ka Shing Faculty of Medicine, The University of Hong Kong, Pok Fu Lam, Hong Kong SAR, China; 4 School of Biomedical Sciences, Li Ka Shing Faculty of Medicine, The University of Hong Kong, Pok Fu Lam, Hong Kong SAR, China; Institute of Biomedicine, Sahlgrenska Academy, University of Gothenburg, SWEDEN

## Abstract

Driver mutations are the genetic variants responsible for oncogenesis, but how specific somatic mutational events arise in cells remains poorly understood. Mutational signatures derive from the frequency of mutated trinucleotides in a given cancer sample, and they provide an avenue for investigating the underlying mutational processes that operate in cancer. Here we analyse somatic mutations from 7,815 cancer exomes from The Cancer Genome Atlas (TCGA) across 26 cancer types. We curate a list of 50 known cancer driver mutations by analysing recurrence in our cohort and annotations of known cancer-associated genes from the Cancer Gene Census, IntOGen database and Cancer Genome Interpreter. We then use these datasets to perform binary univariate logistic regression and establish the statistical relationship between individual driver mutations and known mutational signatures across different cancer types. Our analysis led to the identification of 39 significant associations between driver mutations and mutational signatures *(P* < 0.004, with a false discovery rate of < 5%). We first validate our methodology by establishing statistical links for known and novel associations between driver mutations and the mutational signature arising from *Polymerase Epsilon* proofreading deficiency. We then examine associations between driver mutations and mutational signatures for AID/APOBEC enzyme activity and deficient mismatch repair. We also identify negative associations (odds ratio < 1) between mutational signatures and driver mutations, and here we examine the role of aging and cigarette smoke mutagenesis in the generation of driver mutations in *IDH1* and *KRAS* in brain cancers and lung adenocarcinomas respectively. Our study provides statistical foundations for hypothesised links between otherwise independent biological processes and we uncover previously unexplored relationships between driver mutations and mutagenic processes during cancer development. These associations give insights into how cancers acquire advantageous mutations and can provide direction to guide further mechanistic studies into cancer pathogenesis.

## Author summary

Cancer develops when cells acquire somatic driver mutations that confer a growth advantage. The origins underlying the development of many of these mutations remain largely unknown. Mutational signatures represent the frequency of different somatic mutations across a genome and can be used to characterise the mutational processes that have operated over time within an individual cancer. In this study, we use mutational signatures as a tool to identify associations between mutational processes and cancer-causing mutations known as drivers. We hypothesised that in some cases a driver mutation would be the underlying cause of an associated signature. In other cases, the altered trinucleotide preferences arising from a signature would have increased the likelihood of the associated driver mutation arising. We determine which scenario is most likely to be the case by examining the trinucleotide context of each driver mutation. Here we identify 39 significant associations using a cohort of 7,815 cancer exomes. We examine known and novel associations between driver mutations and mutational signatures arising from processes such as defective proofreading during DNA replication, AID/APOBEC enzyme-associated mutagenesis and deficient mismatch repair. Our study explores important relationships that can inform our understanding of the complex pathogenic history associated with cancer development.

## Introduction

Cancer occurs following the accumulation of somatic mutations within cellular DNA [1]. Somatic mutations can arise as a result of exposure to external DNA damaging agents, or as a consequence of internal errors in DNA replication or repair [2]. Cells undergo malignant transformation following the acquisition of a subset of somatic mutations, termed driver mutations [3]. Driver mutations confer a growth advantage to cells, and subsequently undergo positive selection in a population. Driver mutations typically affect certain cancer-associated genes by, for example, activating an oncogene or inactivating a tumour suppressor gene. Research in recent years has led to the identification of hundreds of driver mutations in cancer-associated genes, but only a handful of driver mutations are sufficient for oncogenesis in a single cancer sample [4]. Driver mutations form in the cancer genome alongside potentially hundreds of thousands of passenger mutations [3]. Passenger mutations are not directly involved in cancer progression and do not confer a selective advantage.

One method developed to characterise the spectrum of mutations that have accumulated in an individual cancer genome is to determine which mutational signatures are present in that sample’s DNA. Mutational signatures are displayed according to six substitution types (C>A, C>G, C>T, T>A, T>C and T>G) in the context of all trinucleotide combinations, thus representing each of the 96 possible mutation frequencies. Thirty mutational signatures have been curated following Alexandrov et al [5] at the ‘Signatures of Mutational Processes in Human Cancer’ website, hosted by the Catalogue of Somatic Mutations in Cancer (COSMIC) database [6, 7]. These signatures reveal which mutational processes have been operative in a cancer genome, as these processes leave a characteristic imprint on the mutational profile [5, 8]. The underlying aetiology of many of these mutational signatures has already been defined. Some signatures have been attributed to defective DNA replication or repair, and others to particular exogenous or endogenous mutagenic processes [5, 8, 9]. The final mutational landscape in any cancer genome will be determined by the strength and exposure duration of each mutational process, which will result in a unique combination of mutational signatures [5, 8].

A recent study suggested that approximately two-thirds of mutations in human cancers arise due to errors in DNA replication occurring over time [10]. Even cancers that have a strong environmental component therefore still harbour mutations incurred by unavoidable DNA replication errors [10]. Relatively little research has focused on investigating which specific driver mutations typically arise due to factors associated with the environment, heritability or DNA replication. We therefore undertook this study to determine the association between common driver mutations and distinct mutational processes in human cancer. Many mutational processes operating in cellular DNA alter the frequencies of mutation accumulation at certain trinucleotide contexts, thus resulting in these definable mutational signatures [5]. We hypothesised that these mutational preferences would increase the likelihood of certain driver mutations arising in the genome, revealing new insights into cancer pathogenesis. We analysed somatic mutations accumulating in the exomes of 7,815 cancer samples from The Cancer Genome Atlas (TCGA), across 26 cancer types. Combining 50 driver mutations with 30 known mutational signatures, we observed 39 significant associations at a false discovery rate (FDR) of < 5%. Many of these associations have not previously been explored, and we reveal relationships that improve our understanding of cancer development. Our findings have the potential to inform new avenues of research into cancer treatment and prevention.

## Results

### Landscape of mutational signatures and driver mutations

Our curation and filtering criteria used to select samples for downstream analysis are detailed in the **Methods** (see also **S1 Fig**). We selected a cohort of 7,815 samples across 26 cancer types for analysis (**Tables 1 and S1**). These samples harboured a median of 100 single nucleotide somatic variants per exome.

**Table 1:** Cancer types, abbreviations and cohort sizes for the whole-exome sequenced samples analysed in this study.

Cancer types	Abbreviation	Number of samples	Fraction of cohort (%)
Adrenocortical carcinoma	ACC	43	0.55
Bladder urothelial carcinoma	BLCA	398	5.09
Breast invasive carcinoma	BRCA	805	10.3
Cervical squamous cell carcinoma and endocervical adenocarcinoma	CESC	296	3.79
Cholangiocarcinoma	CHOL	42	0.54
Colon adenocarcinoma (COAD) and rectum adenocarcinoma (READ)	CRC	553	7.08
Lymphoid neoplasm diffuse large B-cell lymphoma	DLBC	48	0.61
Esophageal carcinoma	ESCA	180	2.3
Glioblastoma multiforme	GBM	362	4.63
Head and neck squamous cell carcinoma	HNSC	490	6.27
Kidney renal clear cell carcinoma	KIRC	290	3.71
Kidney renal papillary cell carcinoma	KIRP	243	3.11
Brain lower grade glioma	LGG	202	2.58
Liver hepatocellular carcinoma	LIHC	355	4.54
Lung adenocarcinoma	LUAD	536	6.86
Lung squamous cell carcinoma	LUSC	487	6.23
Mesothelioma	MESO	46	0.59
Ovarian serous cystadenocarcinoma	OV	420	5.37
Pancreatic adenocarcinoma	PAAD	117	1.5
Prostate adenocarcinoma	PRAD	191	2.44
Sarcoma	SARC	212	2.71
Skin cutaneous melanoma	SKCM	450	5.76
Stomach adenocarcinoma	STAD	418	5.35
Thyroid carcinoma	THCA	52	0.67
Uterine corpus endometrial carcinoma	UCEC	525	6.72
Uterine carcinosarcoma	UCS	54	0.69

We identified mutational signatures in these samples by using the Sigfit [11] R package. We found that mutational signatures in our cohort were comparable with signatures previously defined in similar cohorts [5] (**S2 Fig**). For example, Signature 1 exhibits clock-like properties, and the number of mutations in this signature correlates with age across a majority of cancer types [5, 12]. Signature 1 was the most common mutational signature that we identified, contributing an average of 6.21% toward all mutational signatures measured across our cohort. Considering a mutational signature to be present if it contributes > 5% toward the entire mutational load of a sample, we found signature 1 to be present in 35% of all samples (*n* = 2,750). Signatures 2 and 13 were the next most common mutational signatures that we identified (5.80% and 6.19%, respectively). These signatures are associated with the activity of activation-induced cytosine deaminase (AID) and apolipoprotein B mRNA editing enzyme catalytic polypeptide-like (APOBEC) enzymes [5, 13]. We found signature 2 in 26% (*n* = 2,016 samples) and signature 13 in 30% (*n* = 2,343 samples) of all samples examined.

**Fig 1:**
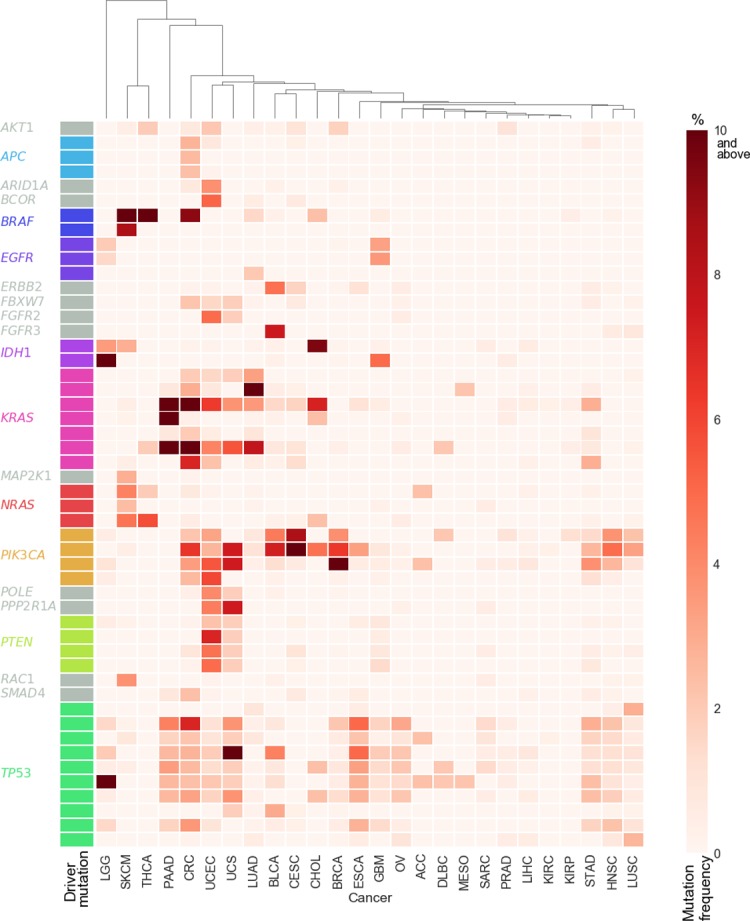
Heat map depicting the frequency of driver mutations within each cancer type analysed. Mutation frequency within each cancer type ranges from light red (0%) to dark red (≥ 10% of samples), with driver genes indicated on the y-axis. Cancer types are clustered across the x-axis. See **Table 1**for the full cancer type name that corresponds to each of the abbreviations listed on the x-axis.

By investigating recurrence of mutations in known cancer driver genes (see **Methods** and **S1 Fig**), we selected 50 driver mutations for potential association with mutational signatures (**S2 Table**). These mutations alter 21 different genes, with *TP53* (*n* = 10), *KRAS* (*n* = 7), *PIK3CA* (*n* = 4) and *PTEN* (*n* = 4) harbouring at total of 50% of all of the driver mutations selected. We next defined the landscape of somatic driver mutations across the cancer samples in our cohort (**Fig 1**). Focusing on the most common driver mutations, we found ten mutations present in > 10% of samples in at least one cancer type. These mutations include *BRAF* p.V600E, which was also the most common driver mutation in our cohort (*n* = 287). The *BRAF* p.V600E mutation was most frequent in skin cutaneous melanoma and thyroid carcinoma, affecting 43% (*n* = 193) and 56% (*n* = 29) of samples respectively. Of the other frequent mutations, four mutations altered *KRAS* amino acid G12, primarily affecting colorectal, lung and pancreatic adenocarcinomas. *PIK3CA* (p.E545K and p.H1047R) and *TP53* (p.R248Q and p.R273C) harboured two highly frequent mutations each, and *IDH1* p.R132H was most frequent in brain lower grade glioma (57%, *n* = 116). Taken together, we found these driver mutations to generally represent known frequencies in other cancer cohorts [3, 14, 15].

### Significant associations between mutational signatures and driver mutations

**Fig 2:**
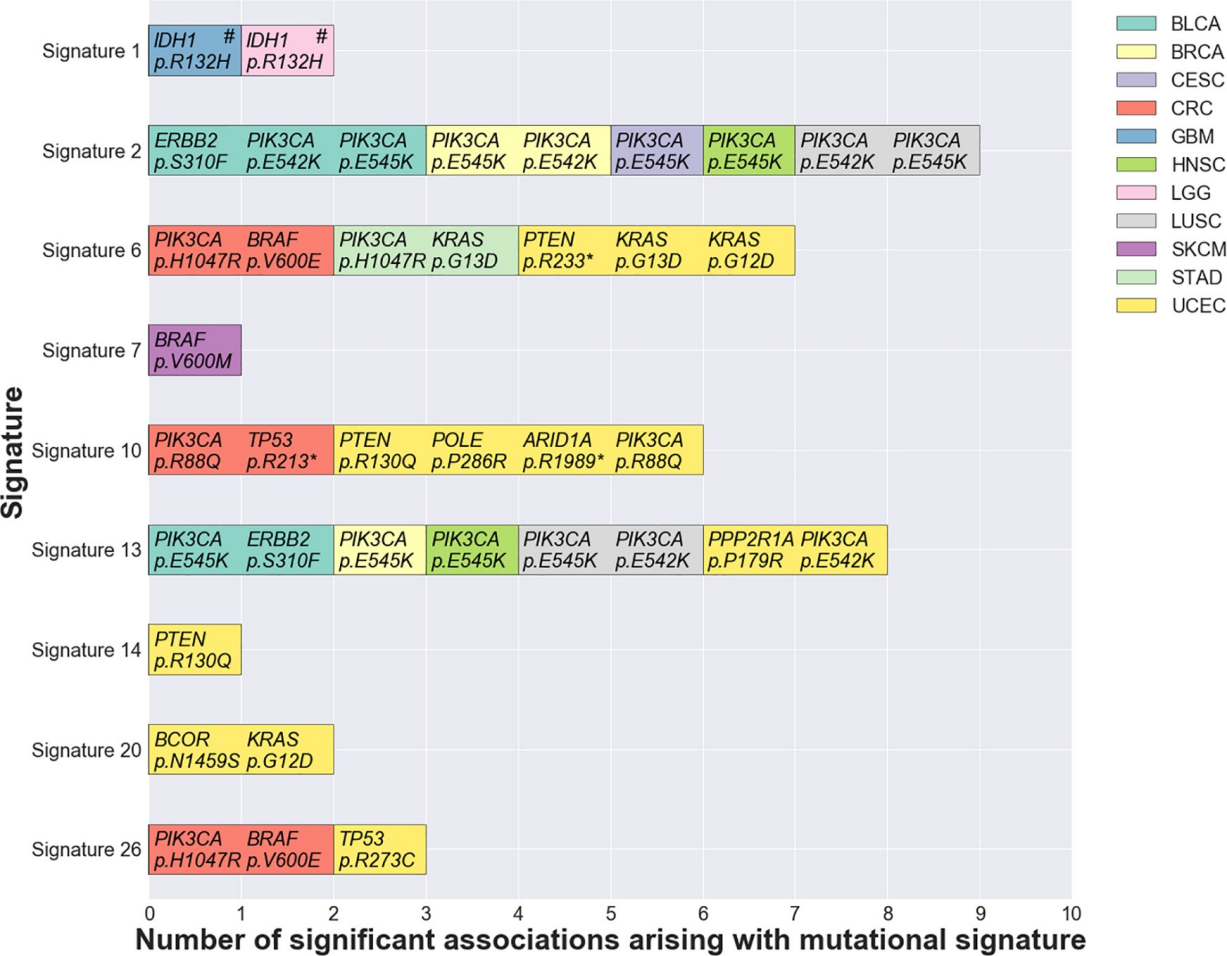
Significant associations between driver mutations and mutational signatures within cancer types. Bars are coloured according to cancer type. Negative associations (odds ratio < 1) are indicated with a hash (#). See **Table 1**for the full cancer type name that corresponds to each of the abbreviations listed in the legend.

After quantifying the landscape of driver mutations and mutational signatures amongst the cancer samples in our cohort, we next sought to investigate whether any driver mutations were significantly associated with the presence or contribution of any mutational signatures. To exclude potentially spurious associations, we only examined associations in which ≥ 10 samples in a given cancer type harboured the driver mutation of interest, and ≥ 10 samples in that same cancer type harboured the signature of interest at a frequency of ≥ 20%. Subsequently, we tested 411 associations for statistical significance (**S3 Table**), examining a total of 15 cancer types and 13 mutational signatures across the 50 driver mutations. We performed a binary univariate logistic regression analysis to test each association and defined significance at *P* < 0.004 (FDR < 5%; see **Methods** and **S3 Fig**). After reciprocal negative associations were excluded (see **Methods**), we found 39 significant associations between driver mutations and mutational signatures (**Table 2**and summary in **Fig 2**). These associations arose across 11 cancer types, affecting 9 mutational signatures and 18 driver mutations from 11 different genes (**Table 2**). We found the highest numbers of significant associations in uterine corpus endometrial carcinoma (*n* = 13) and colorectal adenocarcinoma (*n* = 6; **Table 2**). Of the total of 39 significant associations that we observed, two were significant negative associations (odds ratio < 1; **Table 2**). These negative associations arose between signature 1 and *IDH1* p.R132H in brain lower grade glioma and glioblastoma multiforme (**Table 2**).

**Table 2:** Significant associations between mutational signatures and driver mutations within individual cancer types, alongside proposed aetiology and frequency of the trinucleotide context in the associated mutational signature.

Mutational signature	Proposed aetiology of mutational signature*	Driver mutation	Protein change	Odds ratio (OR)	Cancer type^#^	Mutated samples in cancer type (%)	Trinucleotide context	Frequency of trinucleotide context in signature^^^	
								Amount (%)	Rank
Signature 1	Spontaneous deamination of 5-methylcytosine	*IDH1* c.395G>A	p.R132H	Less than 1	GBM	4.97	A[C>T]G	17.16	High
		*IDH1* c.395G>A	p.R132H	Less than 1	LGG	57.43	A[C>T]G	17.16	High
Signature 2	Activity of the AID/APOBEC family of cytidine deaminases	*PIK3CA* c.1633G>A	p.E545K	Greater than 1	LUSC	3.29	T[C>T]A	41.99	High
		*PIK3CA* c.1633G>A	p.E545K	Greater than 1	HNSC	4.9	T[C>T]A	41.99	High
		*PIK3CA* c.1633G>A	p.E545K	Greater than 1	CESC	14.19	T[C>T]A	41.99	High
		*PIK3CA* c.1633G>A	p.E545K	Greater than 1	BRCA	6.34	T[C>T]A	41.99	High
		*ERBB2* c.929C>T	p.S310F	Greater than 1	BLCA	4.77	T[C>T]C	8.2	High
		*PIK3CA* c.1633G>A	p.E545K	Greater than 1	BLCA	7.29	T[C>T]A	41.99	High
		*PIK3CA* c.1624G>A	p.E542K	Greater than 1	LUSC	2.26	T[C>T]A	41.99	High
		*PIK3CA* c.1624G>A	p.E542K	Greater than 1	BRCA	3.85	T[C>T]A	41.99	High
		*PIK3CA* c.1624G>A	p.E542K	Greater than 1	BLCA	4.52	T[C>T]A	41.99	High
Signature 6	Defective DNA mismatch repair	*PIK3CA* c.3140A>G	p.H1047R	Greater than 1	STAD	3.83	A[T>C]G	2.17	Low
		*KRAS* c.38G>A	p.G13D	Greater than 1	UCEC	2.29	G[C>T]C	7.73	High
		*KRAS* c.38G>A	p.G13D	Greater than 1	STAD	2.87	G[C>T]C	7.73	High
		*KRAS* c.35G>A	p.G12D	Greater than 1	UCEC	6.29	A[C>T]C	1.63	Low
		*BRAF* c.1799T>A	p.V600E	Greater than 1	CRC	9.22	G[T>A]G	0.06	Low
		*PTEN* c.697C>T	p.R233*	Greater than 1	UCEC	4.95	A[C>T]G	9.08	High
		*PIK3CA* c.3140A>G	p.H1047R	Greater than 1	CRC	3.44	A[T>C]G	2.17	Low
Signature 7	Ultraviolet light exposure	*BRAF* c.1798G>A	p.V600M	Greater than 1	SKCM	8.44	A[C>T]T	0.43	Low
Signature 10	Altered activity of the error-prone polymerase *POLE*	*POLE* c.857C>G	p.P286R	Greater than 1	UCEC	4	C[C>G]T	0	Low
		*PIK3CA* c.263G>A	p.R88Q	Greater than 1	UCEC	5.9	T[C>T]G	21.41	High
		*PIK3CA* c.263G>A	p.R88Q	Greater than 1	CRC	2.53	T[C>T]G	21.41	High
		*TP53* c.637C>T	p.R213*	Greater than 1	CRC	2.17	T[C>T]G	21.41	High
		*PTEN* c.389G>A	p.R130Q	Greater than 1	UCEC	4.76	T[C>T]G	21.41	High
		*ARID1A* c.5965C>T	p.R1989*	Greater than 1	UCEC	3.81	T[C>T]G	21.41	High
Signature 13	Activity of the AID/APOBEC family of cytidine deaminases	*PPP2R1A* c.536C>G	p.P179R	Greater than 1	UCEC	4.38	C[C>G]C	0.09	Low
		*PIK3CA* c.1633G>A	p.E545K	Greater than 1	BLCA	7.29	T[C>T]A	11.38	High
		*PIK3CA* c.1633G>A	p.E545K	Greater than 1	HNSC	4.9	T[C>T]A	11.38	High
		*PIK3CA* c.1633G>A	p.E545K	Greater than 1	BRCA	6.34	T[C>T]A	11.38	High
		*PIK3CA* c.1624G>A	p.E542K	Greater than 1	UCEC	3.24	T[C>T]A	11.38	High
		*PIK3CA* c.1624G>A	p.E542K	Greater than 1	LUSC	2.26	T[C>T]A	11.38	High
		*ERBB2* c.929C>T	p.S310F	Greater than 1	BLCA	4.77	T[C>T]C	1.5	Low
		*PIK3CA* c.1633G>A	p.E545K	Greater than 1	LUSC	3.29	T[C>T]A	11.38	High
Signature 14	Loss of mismatch repair and polymerase proofreading	*PTEN* c.389G>A	p.R130Q	Greater than 1	UCEC	4.76	T[C>T]G	0.94	Low
Signature 20	Loss of mismatch repair and polymerase proofreading	*KRAS* c.35G>A	p.G12D	Greater than 1	UCEC	6.29	A[C>T]C	2.22	Low
		*BCOR* c.4376A>G	p.N1459S	Greater than 1	UCEC	5.14	A[T>C]T	0.73	Low
Signature 26	Defective DNA mismatch repair	*PIK3CA* c.3140A>G	p.H1047R	Greater than 1	CRC	3.44	A[T>C]G	5.18	High
		*BRAF* c.1799T>A	p.V600E	Greater than 1	CRC	9.22	G[T>A]G	0.14	Low
		*TP53* c.817C>T	p.R273C	Greater than 1	UCEC	2.1	G[C>T]G	2.25	Low

* Proposed aetiology obtained from refs [5] and [9] and ‘Signatures of Mutational Processes in Human Cancer’ curated by the Catalogue of Somatic Mutations in Cancer (COSMIC) database [6, 7];

^#^ List of abbreviations for each cancer type are given in **Table 1**;

^^^ Trinucleotide frequencies for mutational signatures are obtained from ‘Signatures of Mutational Processes in Human Cancer’ curated by the COSMIC database [6, 7]. Amount designates the percentage of all mutations in the signature that occur in the trinucleotide context of the specific driver mutation. The rank designates whether that mutation in its trinucleotide context is high (amount > 5%) or low (amount ≤ 5%) in the mutational signature.

We validated a subset of our findings using an independent whole-exome sequenced cancer cohort of 619 colorectal cancer samples published in [16]. Using somatic mutations from this cohort, we examined the statistical relationship between mutational signatures and driver mutations for each of the six associations that we found to be significant in colorectal cancer in our existing analysis using TCGA data (**Table 2**). Validating these colorectal cancer results from our current study with this independent cohort, we found 5 of the 6 tested associations to be significant at *P* = 0.0072 or below (**S4 Table**).

### Acquisition of driver mutations associated with signature 10

**Fig 3:**
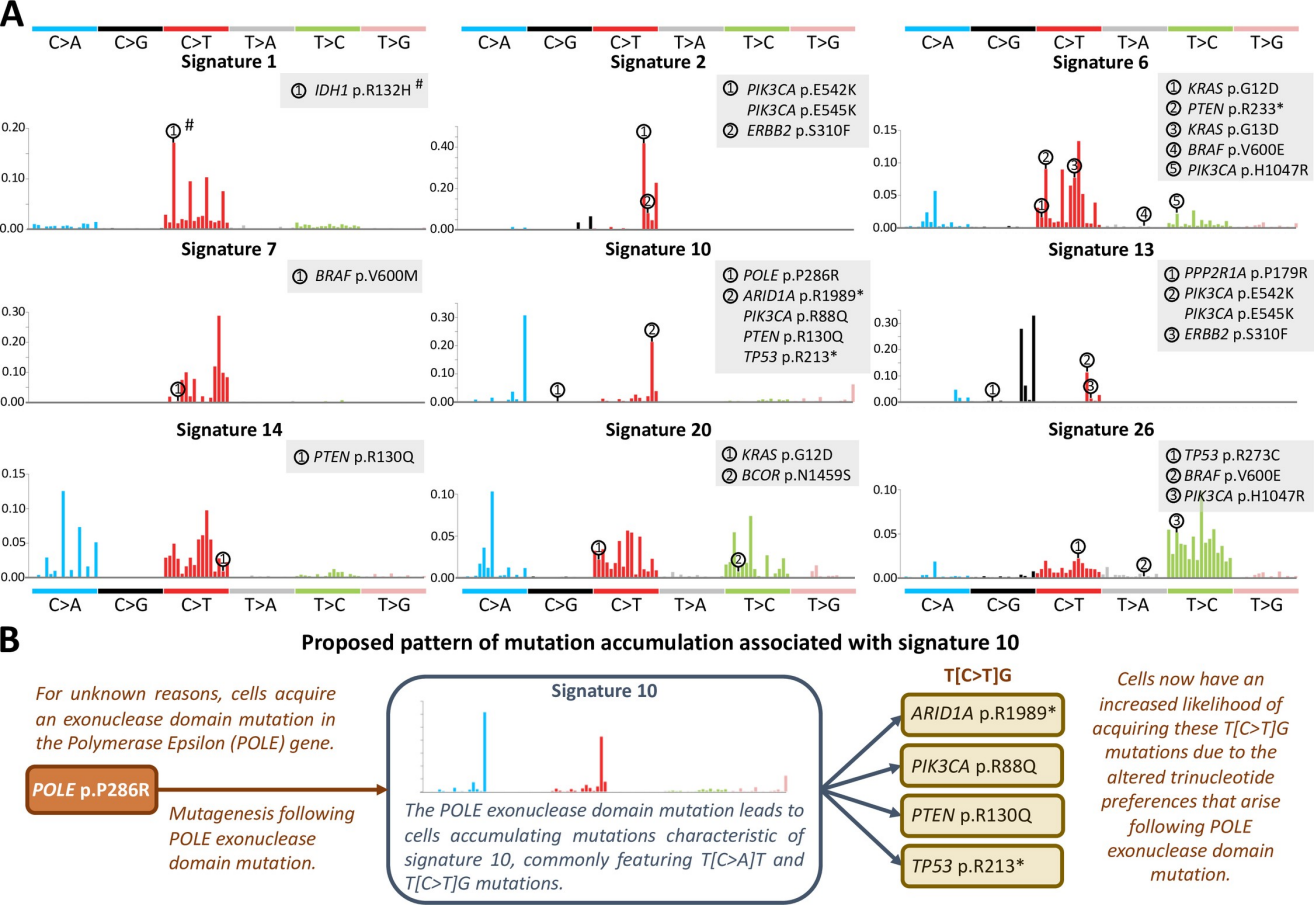
Trinucleotide context of driver mutations that are significantly associated with mutational signatures, and schematic depicting proposed mutation accumulation associated with signature 10. **(A)** The trinucleotide contexts of significantly associated driver mutation are indicated by numbering on each signature plot. Mutational signature images were generated using the Sigfit [11] R package. Negative associations (odds ratio < 1) are indicated with a hash (#). **(B)** Schematic diagram depicting the proposed mechanism of driver mutation accumulation in *Polymerase Epsilon (POLE)* exonuclease domain mutated cancers harbouring signature 10.

To validate our methodology, we first investigated the six significant associations that we observed between driver mutations and signature 10 across uterine corpus endometrial carcinoma and colorectal adenocarcinoma (**Table 2**). Here, existing literature allows us to validate many of the patterns of causality inferred from our regression results. Signature 10 arises in cancers which harbour *Polymerase Epsilon (POLE)* exonuclease domain mutations. In our study, we found the *POLE* p.P286R (c.857C>G) mutation to be significantly associated with signature 10 in uterine corpus endometrial carcinoma (**Table 2**). The trinucleotide context of this mutation (C[C>G]T) is not frequently observed in signature 10 (**Fig 3A**), supporting existing literature that demonstrates the *POLE* p.P286R mutation to underlie many instances of the presence of signature 10 in cancer [5, 17]. The remaining driver mutations that we found to be significantly associated with signature 10 (*PIK3CA* p.R88Q, *PTEN* p.R130Q, *ARID1A* p.R1989* and *TP53* p.R213*) all occur in a T[C>T]G context (**Table 2**). This trinucleotide context is frequently mutated in signature 10 (**Fig 3A**), suggesting that these driver mutations likely arose as a direct result of exposure to the mutagenic processes underlying this signature. In fact, in colorectal cancer, *TP53* p.R213* mutations have been suggested to arise in response to *POLE* exonuclease domain mutation, where DNA methylation at this CpG trinucleotide may further enhance the likelihood of mutation occurrence [18]. Endometrial cancers with *POLE* exonuclease domain mutations have also been shown to exhibit a high prevalence of *TP53*, *ARID1A*, *PTEN* and *PIK3CA* mutations [19]. By analysing the results of our study in this way, we can demonstrate a likely pathway for oncogenesis via driver mutation accumulation associated with signature 10. This pattern is demonstrated schematically in **Fig 3B**, and it serves as an example for the associations that we subsequently explore in this study.

### AID/APOBEC-associated mutagenesis associated with signatures 2 and 13

We found that a striking 36% (*n* = 14) of the associations that we identified arose between driver mutations in *PIK3CA* and signatures 2 or 13 across six different cancer types. (**Table 2**). Signatures 2 and 13 are attributed to the action of the AID/APOBEC family of cytidine deaminases [5, 13]. APOBEC activity has been implicated in the generation of specific *PIK3CA* mutations at p.E542K (c.1624G>A) and p.E545K (c.1633G>A) [20], and we identified associations with both mutations in our study. Both mutations occur in the T[C>T]A context that is frequently mutated in signatures 2 and 13 (**Table 2 and Fig 3A**). In fact, both of these mutations match the extended context of the [T/C]TC[A/G] motif which has been established for APOBEC3A binding in single-stranded DNA (ssDNA) [21]. In addition to the *PIK3CA* mutations, we found signatures 2 and 13 to be associated with *ERBB2* p.S310F (c.929C>T) mutation in bladder cancer, and signature 13 to be associated with *PPP2R1A* p.P179R (c.536C>G) mutation in uterine corpus endometrial carcinoma (**Table 2**). The broader contexts of these mutations, T[C>T]C and C[C>G]C respectively, do not match well with the typical APOBEC3A/B mutational context. However, APOBEC enzyme mutagenesis has also been shown to preferentially accumulate on ssDNA associated with lagging strand replication, and with transcription bubbles [22–24]. In addition, biophysical studies have shown that APOBEC enzymes also preferentially bind cytosines that are within ssDNA stem-loops [21, 25]. Using ssDNA folding predictions (see **Methods**), we find that both of the cytosines mutated in the *ERBB2* and *PPP2R1A* drivers are predicted to be located at stem-loops, and that these loops are greater than three bases in size (**S4 Fig**). Loops with a size greater than three bases have been shown to aid APOBEC enzyme binding [25], with the APOBEC3A binding site requiring bent ssDNA [21]. Confirmation of whether and how APOBEC3A/B binds and mutates these DNA sequences will need to be experimentally validated. However, the association with ssDNA and potential stem loop formation provides one possible explanation that may account for the association of signatures 2 and 13 with these mutations.

### Acquisition of driver mutations associated with signatures for mismatch repair deficiency

We found 13 significant associations arising between driver mutations and mutational signatures implicated in mismatch repair deficiency (signatures 6, 14, 20 and 26 [5, 9]; **Table 2**). These associations arise in stomach and colorectal adenocarcinoma and uterine corpus endometrial carcinoma, and they involve eight different driver mutations (**Table 2**). Interestingly, only three of these eight mutations occur in trinucleotide contexts that frequently appear in any of the mismatch repair deficiency signatures (**Table 2**). Among the remaining five mutations is *BRAF* p.V600E, which is significantly associated with signatures 6 and 26 in colorectal adenocarcinoma (**Table 2**). Signatures 6 and 26 are the only mutational signatures with significant associations that represent mismatch repair deficiency alone [5, 9]. The mechanism underlying the association between *BRAF* p.V600E and mismatch repair deficiency has not yet been established to our knowledge. It is possible that this association arises because acquisition of a *BRAF* p.V600E mutation predisposes otherwise normal cells to developing mismatch repair deficiency, though this hypothesis requires further investigation. In support of this hypothesis, *BRAF* p.V600E mutations do occur much less commonly in hereditary nonpolyposis colorectal cancers [26]–cancers which frequently arise due to germline mismatch repair defects. Our results suggest that many driver mutations in cancers with mismatch repair deficiencies may arise independently from, or prior to, loss of mismatch repair. However, we note that our analyses cannot eliminate the possibility that we have observed certain significant associations because loss of mismatch repair increases the overall mutation rate in a given cancer genome, regardless of trinucleotide mutation context. Therefore, despite these mutations less frequently arising as a result of mismatch repair defects due to their specific trinucleotide contexts, they may become significantly associated either due to an altered rate of mutation accumulation in mismatch repair deficient cancers or because they confer an overwhelming selective advantage whenever they do occur.

### Significant negative associations and inference to causality

**Fig 4:**
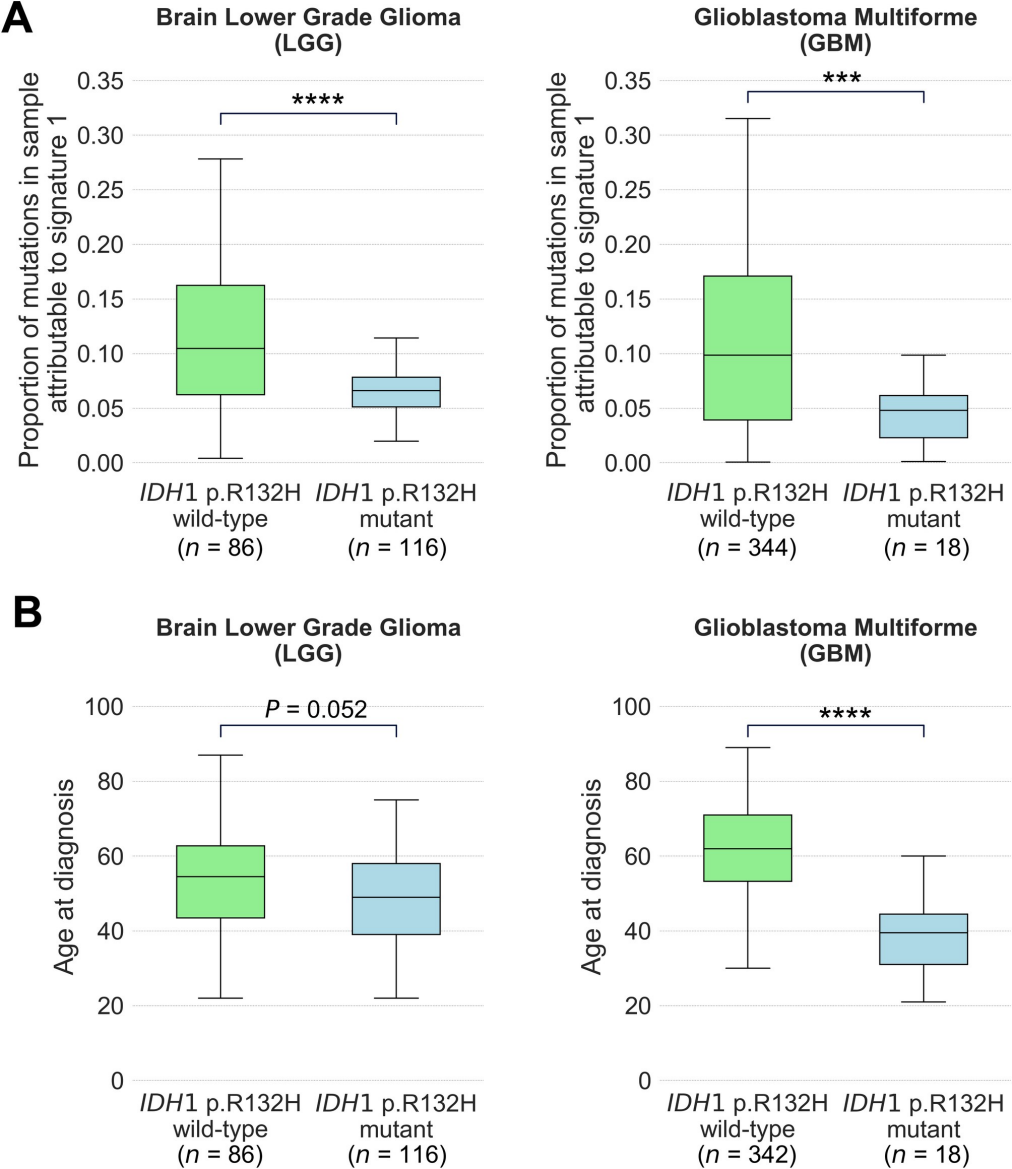
Associations of *IDH1* p.R132H driver mutation with mutational signature 1 and with age at diagnosis of people with brain cancers. **(A)** Proportion of mutations attributable to signature 1 and **(B)** age at diagnosis of people with tumours that are wild-type and mutant for *IDH1* p.R132H in brain lower grade glioma (LGG; left) and glioblastoma multiforme (GBM; right). Box plots show median and quartiles, with significance by two-sided Mann Whitney U-Test in **(A**) and unpaired t-test in **(B)**. **** denotes *P* < 0.0001 and *** denotes *P* < 0.001.

Our analyses identified two significant negative associations (odds ratio < 1) where we found no reciprocal positive associations (see **Methods**). These negative associations arose between the *IDH1* p.R132H driver mutation and signature 1, occurring in brain lower grade glioma and in glioblastoma multiforme (**Table 2**). Demonstrating this negative association, we observed a significantly lower proportion of signature 1 mutations in *IDH1* p.R132H mutant rather than wild-type brain lower grade glioma (*P* < 0.0001) and glioblastoma multiforme (*P* < 0.001; **Fig 4A**) by two-sided Mann Whitney U-Test. Signature 1 is ubiquitous amongst all cancer types and results from a common mutational process associated with increasing age [5, 12]. Consistent with our observed association, we found that patients with *IDH1* p.R132H mutated tumours in our cohort were generally younger than patients with *IDH1* p.R132H wild-type tumours. This association was significant in glioblastoma multiforme (P < 0.0001) and was approaching significance in brain lower grade glioma (*P* = 0.052 by unpaired t-test; **Fig 4B**). *IDH1* mutations have been found to less commonly occur in older people with glioblastoma [27, 28], and the results of our mutational signature analyses provide molecular support for this finding in glioblastoma multiforme and brain lower grade glioma. While age would increase the likelihood of any mutation arising by chance alone, our results suggest that age might disproportionately favour the occurrence of mutations other than *IDH1* p.R132H in these brain cancers, or that this mutation confers a greater selective advantage in younger people. In older people, brain cancers may develop via a pathway that is activated by other mutational events that are age- or replication-associated.

**Fig 5:**
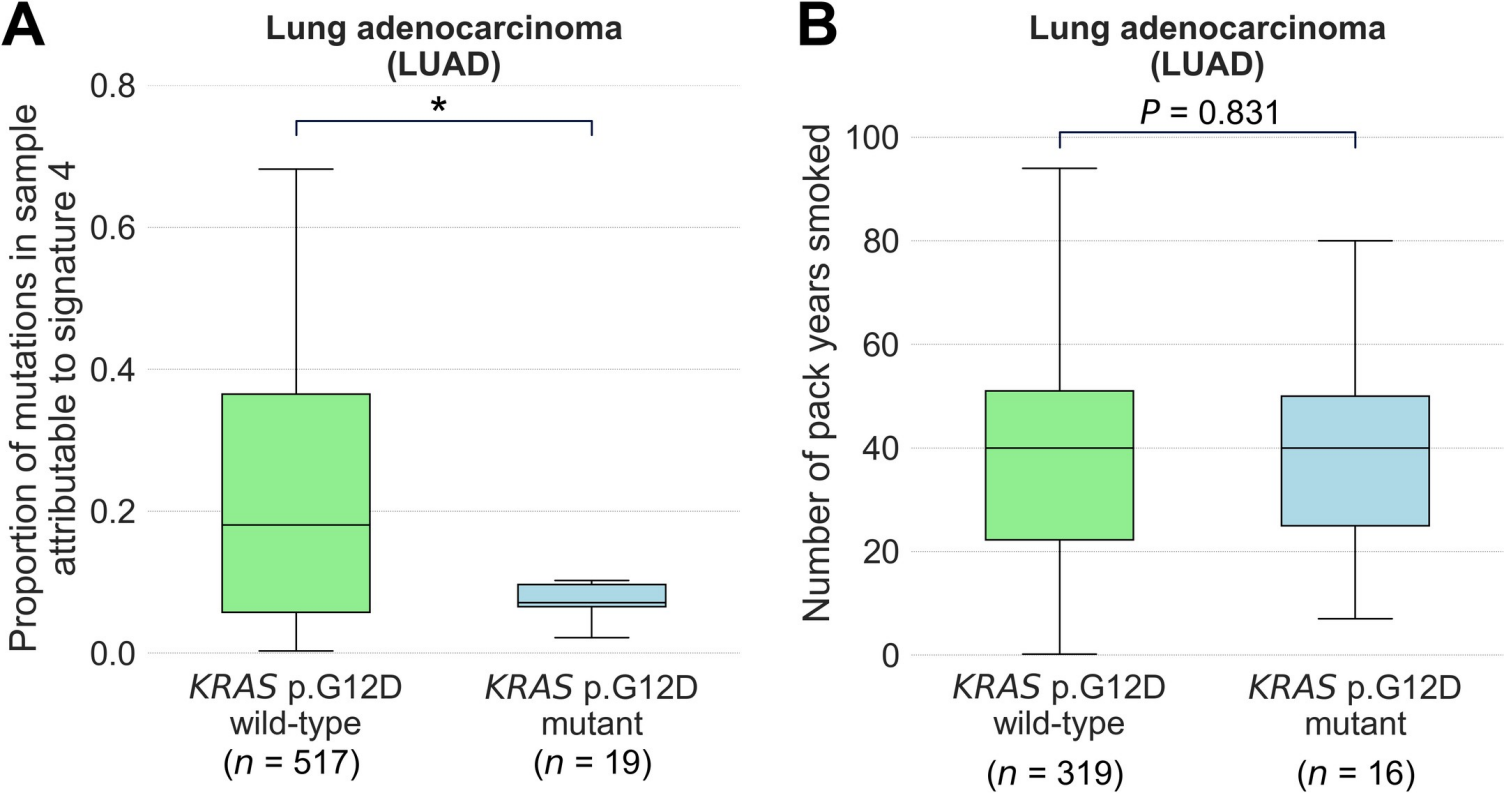
Association of *KRAS* p.G12D driver mutation with mutational signature 4 and with pack years smoked by people with lung adenocarcinoma. **(A)** Proportion of mutations attributable to signature 4 in lung adenocarcinoma (LUAD) tumours that are wild-type and mutant for *KRAS* p.G12D. **(B)** Number of pack years smoked by people with LUAD who have a previous history of smoking, in tumours that are wild-type and mutant for *KRAS* p.G12D. Box plots show median and quartiles, with significance by two-sided Mann Whitney U-Test in **(A**) and unpaired t-test in **(B)**. * denotes *P* < 0.05.

In our analysis, we noted a negative association between signature 4 and *KRAS* p.G12D (c.35G>A) in lung adenocarcinoma where *P* = 0.0075 (**S3 Table**), which is just above our threshold for defining significance (P < 0.004). *KRAS* p.G12D transition mutations are the most common *KRAS* somatic mutation arising in the lung adenocarcinomas of people who have never smoked [29]. Further, people who have never smoked are more likely to have *KRAS* p.G12D mutations in their lung cancers than are people who have smoked [30]. Signature 4 is associated with exposure to the mutagens in cigarette smoke [5], and for these reasons we examined this observed association in more detail. We found that the association we observed by regression was significant at *P* < 0.05 by two-sided Mann Whitney U-Test (**Fig 5A**). Interestingly, in lung adenocarcinomas from people with a recorded history of smoking, we observed no significant difference between the number of pack years smoked by those with and without *KRAS* p.G12D mutations (*P* = 0.831 by unpaired t-test; **Fig 5B**). Our results suggest that increasing proportions of signature 4 mutations do not significantly impact on the likelihood of a lung adenocarcinoma acquiring specifically a *KRAS* p.G12D mutation.

## Discussion

### Many driver mutations arise from accelerated replication-associated errors in cancer

It has been suggested that approximately two-thirds of mutations in cancer arise from DNA replication errors [10]. The results of our study suggest that the altered trinucleotide mutation preferences conferred by replication-associated mutational signatures can skew the odds in favour of the acquisition of certain driver mutations (see **Fig 3B**). 36 of the 37 significant positive associations (odds ratio > 1) that we found in our study are between driver mutations and mutational signatures 2 and 13 (deamination by APOBEC enzymes, which can occur during replication), 6 and 26 (deficient mismatch repair during replication), 10 (proofreading deficiency by *POLE* during replication) and 14 and 20 (deficient mismatch repair and proofreading by *POLE*). 24 of the 36 associations (66%) arising across cancer types occur in cases where the trinucleotide context of the driver mutation frequently arises in the associated signature, implying a possibly direct causal relationship between defective DNA replication and occurrence of that driver mutation. In order for these mutational signatures to become apparent in a genome however, normal replication must have become disrupted, such that the process has accelerated within cancer cells to generate that mutational signature. Some of the mutational signatures that we analyse in this study can arise due to existing germline defects (for example, germline mutations in mismatch repair genes or replicative polymerases) [31]. Similarly, it is possible that somatic events that are causal in generating a replication-associated mutational signature arise as a result of exogenous environmental mutagens (for example, APOBEC enzyme activity could be altered following viral infection [20]). In these circumstances, it could be argued that the hereditary or environmental defect was ultimately responsible for the generation of the specific driver mutations that arose due to the defective DNA replication. Thus, the acquisition of many common driver mutations may be the result of a complex pathogenic history originally arising from non-replication associated effects.

### Associations between BRAF mutations and mutational signatures in cancer

Ultraviolet (UV) radiation has long been epidemiologically associated with the development of melanoma. Mutagenesis associated with signature 7 is directly related to exposure of cells to UV light [5]. We observed a significant association in our study between *BRAF* p.V600M (c.1798G>A) and signature 7 in skin cutaneous melanoma (**Table 2**). While the A[C>T]T trinucleotide context of the *BRAF* p.V600M mutation is infrequent (0.4%) within signature 7, C>T transition mutations within a pyrimidine dimer context do generally characterise this signature [5]. Of note, *BRAF* p.V600E mutations more commonly arise than p.V600M mutations in melanomas [32]. The *BRAF* p.V600E (c.1799T>A) mutation is not a characteristic C>T transition and various models have been proposed for how such mutations may result from exposure to UV radiation [33, 34]. Interestingly, we did not find a significant association between signature 7 and *BRAF* p.V600E in our study (*P* = 0.7086, **S3 Table**). Melanomas arising on skin without chronic sun-induced damage often harbour *BRAF* p.V600E mutations, while those arising on skin with chronic sun-induced damage typically harbour other *BRAF* mutations [35]. Additionally, *BRAF* p.V600E mutations are commonly found in tumours from non-sun-exposed tissues such as thyroid and colorectal cancers, demonstrating that this mutation can arise following mutagenic processes other than UV radiation exposure. In some melanomas, and particularly those with a low contribution from signature 7, we suggest that *BRAF* p.V600E mutations may also arise independently from UV radiation-associated mutagenesis. We note the possibility though, that some *BRAF* p.V600E mutations do arise as a result of mutagenesis following UV radiation exposure, and that this mutation could then confer a particularly strong selective advantage over other pyrimidine dimer-associated mutations, accounting for its observed recurrence in melanoma.

### Relationship to recent published study

During the preparation of our manuscript we became aware of the publication of a similar analysis [36]. In this study, Temko et al made use of TCGA exome sequencing data, together with data from the International Cancer Genome Consortium (ICGC) for cancer driver and mutational signature analysis. The authors used one-sided Mann Whitney U-Tests to examine a selection of potentially correlated driver mutations and mutational signatures, discovering a total of 56 unique significant associations across two analysis approaches. Our study applied different filtering criteria to those of Temko et al when selecting which samples and driver mutations to examine. Hence, in our study only 19 of those 56 associations were tested, of which we found 16 of these 19 tested associations to be significant in our cohort. Conversely, 23 of the 39 significant associations in our study were not identified by Temko et al. Further examination of their analysis reveals that they did not identify these associations due to the association not being tested (8 associations), Temko et al specifically searching for positive associations (2 associations) and different samples and signatures being used when testing for an association (13 associations). Of the latter 13 associations, 11 remained significant at *P* < 0.05 and 5 remained significant at *P* < 0.004 when we conducted a one-sided Mann Whitney U-Test using our cohort (results of one-sided Mann Whitney U-Test shown for all associations in **S3 Table**). Thus, the major differences in the two studies can be accounted for by the different statistical and biological assumptions that we incorporated into our analyses, as well as by considering different sample set and mutational signature identification approaches. Despite this, the similarities in the results attained by both studies provides validation for our collective findings. The differing criteria applied in selection of samples, driver mutations, biological and statistical assumptions ultimately leads to complementary results which collectively provide a more complete list of true significant associations.

### Conclusion

In summary, we performed binary univariate logistic regression analyses to establish a statistical relationship between driver mutations and mutational signatures in 7,815 cancer samples across 26 cancer types. Our analysis led to the identification of 39 significant associations between driver mutations and mutational signatures (*P* < 0.004, FDR < 5%). Our study provides statistical foundations for hypothesised links between otherwise independent biological processes and explores relationships arising between driver mutations and mutagenic processes during cancer formation. These associations provide new insights into how some cancers acquire advantageous mutations and can provide direction to guide further mechanistic studies into mutational processes and cancer development.

## Methods

### Ethics statement

This study was approved by the University of New South Wales (UNSW) Human Research Ethics Advisory Panel (approval no. HC17187). This study analysed data generated by TCGA, which were collected from patients with written informed consent (https://cancergenome.nih.gov/abouttcga/policies/informedconsent).

### Data sources

Somatic mutations from whole exome sequencing data (generated by MuTect [37] and aligned to the GRCh38 reference genome) were downloaded from TCGA via the Genomic Data Commons (GDC) data portal [38] for 10,539 cancer samples. These mutations were used to define mutational signatures and to determine which driver mutations to incorporate into regression analyses. **S1 Fig** presents a flow chart that accompanies this methods description.

Age at diagnosis [“age_at_initial_pathologic_diagnosis”] and number of pack years smoked [“number_pack_years_smoked”] were obtained for relevant cancer types from the UCSC Xena Browser [39]. Samples with no data recorded in these fields were excluded from plots at **Figs 4**and **5**.

### Detection of mutational signatures

To select which samples to include in regression analyses, we excluded any mutations that were annotated by MuTect [37] as present in a ‘panel of normals’, and then kept only cancer samples that harboured ≥ 30 single nucleotide somatic variants in cancer types with ≥ 40 samples. Next, where multiple samples existed for a single patient, we randomly selected only one sample per patient to retain, and we merged TCGA-COAD and TCGA-READ into a single colorectal adenocarcinoma (CRC) cancer type. Our final sample list contained 7,815 samples from 26 cancer types (**Tables 1**and **S1**). We then applied Sigfit (version 1.2.0) [11] R package to determine the proportion of mutations attributable to each of the 30 mutational signatures from the COSMIC [6, 7] ‘Signatures of Mutational Processes in Human Cancer’ database. Sigfit was run in the ‘fit_signatures’ mode using exome-normalised COSMIC signatures and default parameters. To validate the accuracy of our signature generation by Sigfit, we also generated mutational signatures using the DeconstructSigs [40] R package. DeconstructSigs was run using COSMIC signatures, with normalisation via ‘exome2genome’ and a maximum of five signatures allowed per sample. We compared mutational signature proportions for each sample, as generated by both Sigfit and DeconstructSigs, for the nine signatures for which we identified significant associations with driver mutations (**S5 Fig**). We determined the Pearson’s correlation for each signature, finding an average Pearson’s correlation of 0.84 across the nine mutational signatures.

### Selection of driver mutations

To ensure that we did not erroneously exclude any true driver mutations, we used MuTect [37] mutation calls without the ‘panel of normals’ filter when selecting driver mutations. We first selected only missense and stop-gain variants that were present in > 3.5% of samples in at least one cancer type (where TCGA-COAD and TCGA-READ were considered collectively as CRC). Next, we retained only mutations that altered genes listed in the COSMIC ‘Cancer Gene Census’ (Tier 1; retrieved 24 November 2017) [6, 7, 41]. Using only the 7,815 samples described above, we then selected mutations that were present in > 10 samples in at least one cancer type, resulting in a list of 34 driver mutations. We next obtained additional driver mutations from the ‘Catalog of driver mutations’ curated by Integrative Onco Genomics (IntOGen) [42] (retrieved 24 November 2017). We excluded any insertions and deletions as these cannot be directly associated with standard trinucleotide mutational contexts, and any splice site variants as these may not be uniformly captured by exome sequencing. We then selected only mutations present in ≥ 5 samples within the IntOGen database, and > 10 samples from at least one cancer type from our TCGA cohort. This analysis resulted in a list of 47 driver mutations. We merged these two lists of mutations and analysed each using the Cancer Genome Interpreter [43]. We removed any mutations that were not designated as being a tumour driver by the Cancer Genome Interpreter [43]. Our analysis produced a final list of 50 unique driver mutations affecting 21 different genes (**S2 Table**). The trinucleotide contexts of each driver mutation were obtained using BEDTools [44].

### Regression analyses

We applied a binary univariate logistic regression model using the glm package in R to evaluate associations between mutational signatures and driver mutations within each cancer type. The regression model used the following formula, where x represents the proportion of mutations attributed to a given mutational signature in a sample, $p\left(x\right)$ represents the probability that a given driver mutation is present or absent in that sample and β values denote estimates from logistic regression:

$$p\left(x\right)=\frac{1}{1+e^{-\left(\beta_{0}+\beta_{1}x\right)}}$$

The odds ratio was calculated by exponentiating the β_1_ coefficient estimated from the logistic regression model. To limit false discoveries and spurious significant associations arising from limited sample size, we did not test any associations between mutational signatures and driver mutations in cancer types with < 10 samples harbouring either the relevant driver mutation or < 10 samples harbouring ≥ 20% of mutations attributable to that mutational signature. To determine the *P*-value required for FDR < 5%, we performed a randomisation test by randomising driver mutations across samples. For each association for which we tested significance, we randomly shuffled the presence of the driver mutation across samples within that cancer type and then conducted our regression modelling again. We repeated this process across 1,000 iterations, and calculated the mean number of significant associations that we identified (**S3A Fig**). We note that the *P*-values obtained from our regression analyses differ from those obtained following randomisation, with the former including a number of associations that satisfy the alternative hypothesis (**S3B Fig**). At *P* < 0.004, we found that the FDR from these randomised iterations was < 5%. We observed that a significant positive association (odds ratio > 1) between a driver mutation and mutational signature was often accompanied by reciprocal negative associations (odds ratio < 1) between the driver mutation and other mutational signatures tested in that cancer type. Many of these associations are likely to arise due to positively associated signatures necessarily reducing the percentage contribution of all other mutational signatures present in affected samples. For this reason, we excluded any reciprocal negative associations when counting significant associations arising in our regression modelling using actual data, and in our randomisation analysis when establishing FDR.

For validation of a subset of our findings, we obtained single nucleotide somatic mutations for an independent cohort of 619 whole-exome sequenced colorectal cancers from a previously published study [16]. We detected mutational signatures using Sigfit and identified significant associations by logistic regression as described for the TCGA cohort.

### Prediction of ssDNA secondary structure

DNA sequences ± 20 bp of the mutation site in *ERBB2* p.S310F and *PPP2R1A* p.P179R were obtained from the UCSC genome browser. This choice of sequence length was based on an approximation of the size of the remaining single stranded lagging strand template prior to the completion of an Okazaki fragment [45]. To estimate the ssDNA secondary structure, the mFold tool (version 3.6) [46] was used with default parameters.

## Supporting information

S1 Table Cancer samples analysed in this study, along with cancer type and designation of use in regression analysis. (XLSX)

S2 Table Driver mutations analysed in this study, curated as described in Methods and S1 Fig. (XLSX)

S3 Table Associations that were tested for significance, together with results from binary univariate logistic regression and one-sided Mann Whitney U-Test. (XLSX)

S4 Table Results of regression analysis using an independent cohort of 619 whole-exome sequenced colorectal cancers. (XLSX)

S1 Fig Flow chart depicting the methodology applied to identify significant associations between mutational signatures and cancer driver mutations in this study. See **Methods** for further details.
(TIF)

S2 Fig Heat map depicting the proportion of each mutational signature arising within cancers analysed. The proportion of mutations attributable to each mutational signature within individual samples for each cancer type, ranging from light red (0%) to dark red (≥ 30% of mutations attributable to mutational signature). Mutational signatures are clustered across the y-axis. Cancer types are named and coloured along the x-axis. See **Table 1**for the full cancer type name corresponding to each of the abbreviations.
(TIF)

S3 Fig Randomisation analysis for false discovery rate and significance evaluation. **(A)** Proportion of significant results at *P* < 0.004 obtained from 1,000 iterations of randomly shuffled driver mutations within each cancer type. Bars indicate the proportion from 1,000 iterations that each count of significant associations was observed (see **Methods**), with the number found using actual data indicated by a dotted line. **(B)** Frequency at which *P*-values were observed from binary univariate logistic regression of 411 associations using actual (red) and one instance of randomly shuffled mutations (blue).
(TIF)

S4 Fig Predicted single stranded DNA (ssDNA) secondary structure for selected driver mutations. Predicted ssDNA secondary structure shown for **(A)** *PPP2R1A* p.P179R and **(B)** *ERBB2* p.S310F mutations. The mutated base is denoted by the mutation label. Predictions were made using the mFold prediction tool [46] with default parameters.
(TIF)

S5 Fig Mutational signatures generated by Sigfit and DeconstructSigs. The proportion of mutations attributed to each mutational signature is shown for Sigfit (y-axis) and DeconstructSigs (x-axis), where dots indicate individual samples. The Pearson’s correlation (*r*) is indicated for each signature on individual plots.
(TIF)

S1 Data The data underlying the generation of all figures in the manuscript is available as a supplementary file. (ZIP)
