# Supplementary figures and images for: Analysis of 7,815 cancer exomes reveals associations between mutational processes and somatic driver mutations
Source: PLoS Genet. 2018 Nov 9;14(11):e1007779. doi: 10.1371/journal.pgen.1007779 (PMC6249022; doi:10.1371/journal.pgen.1007779)

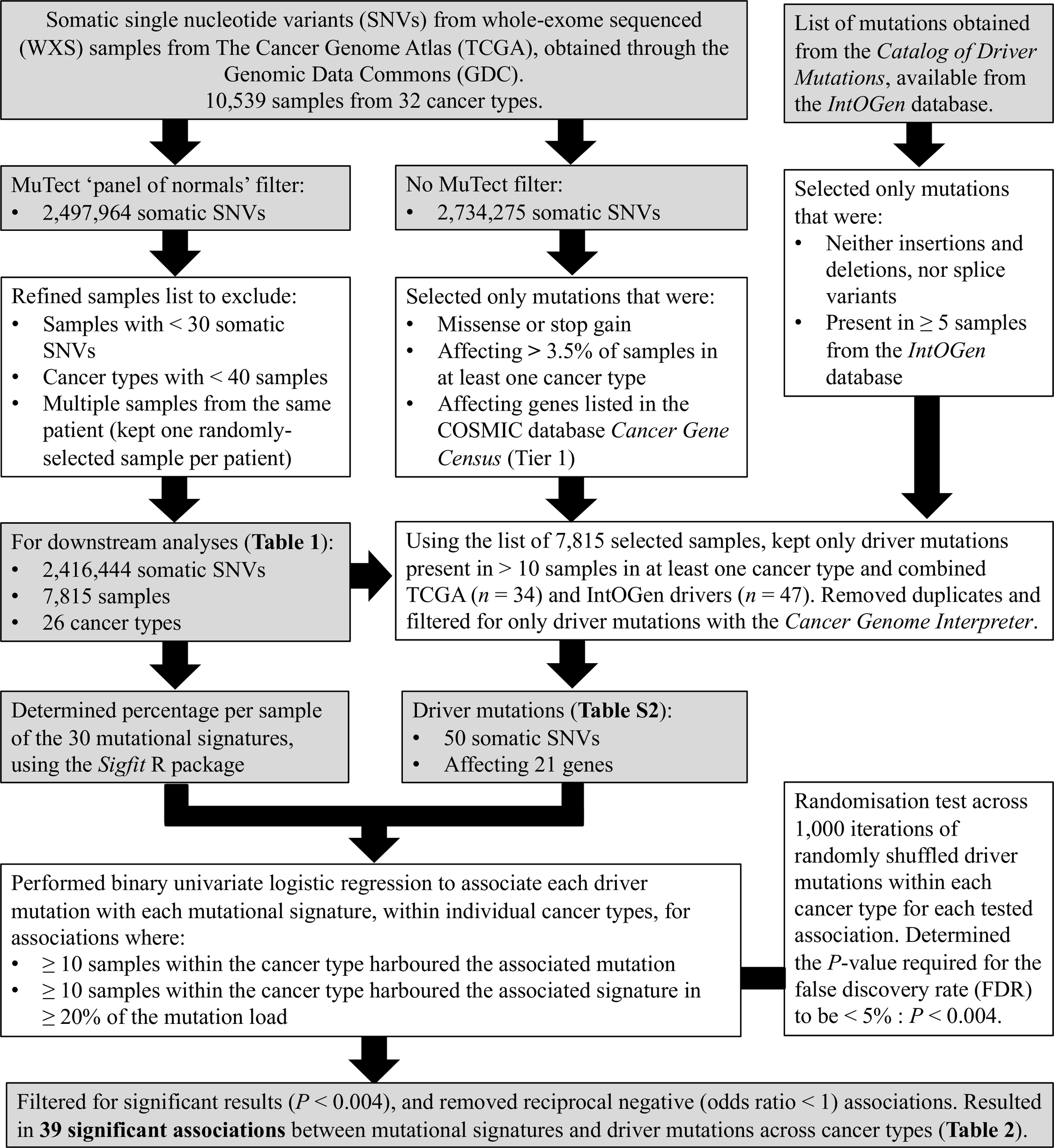

Supplement: S1 Fig — See Methods for further details. (TIF) [file pgen.1007779.s005.tif]

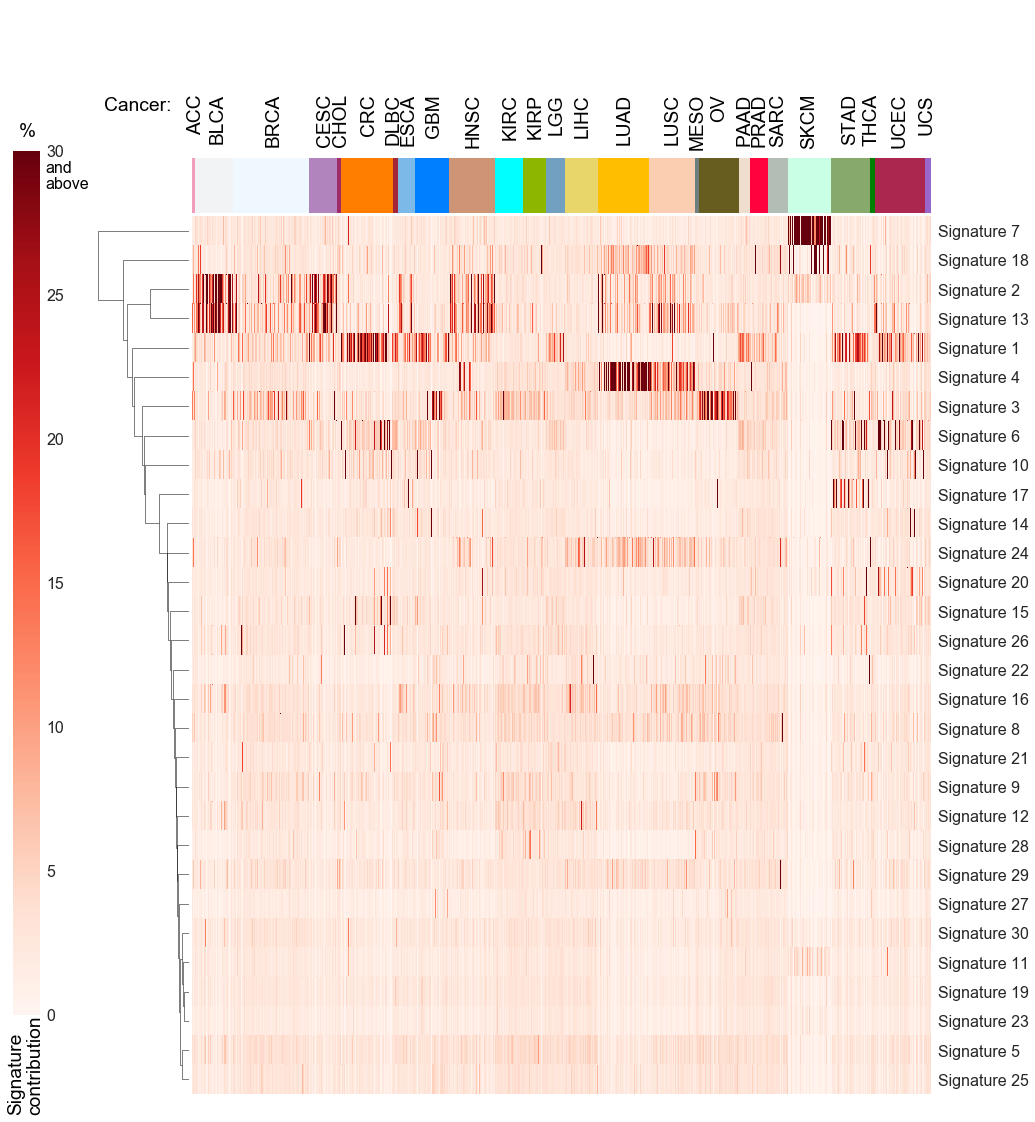

Supplement: S2 Fig — The proportion of mutations attributable to each mutational signature within individual samples for each cancer type, ranging from light red (0%) to dark red (≥ 30% of mutations attributable to mutational signature). Mutational signatures are clustered across the y-axis. Cancer types are named and coloured along the x-axis. See Table 1for the full cancer type name corresponding to each of the abbreviations. (TIF) [file pgen.1007779.s006.tif]

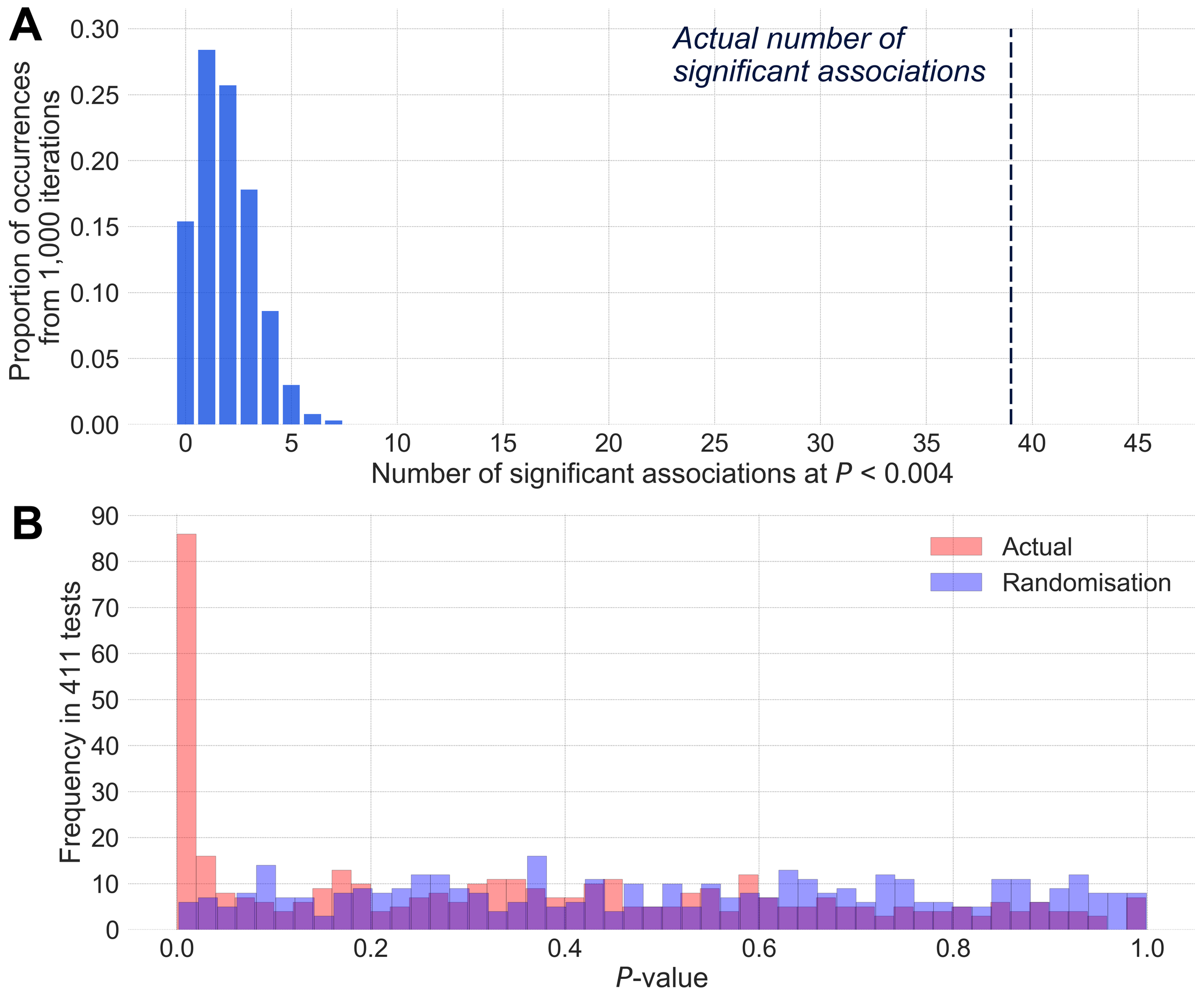

Supplement: S3 Fig — (A) Proportion of significant results at P < 0.004 obtained from 1,000 iterations of randomly shuffled driver mutations within each cancer type. Bars indicate the proportion from 1,000 iterations that each count of significant associations was observed (see Methods), with the number found using actual data indicated by a dotted line. (B) Frequency at which P-values were observed from binary univariate logistic regression of 411 associations using actual (red) and one instance of randomly shuffled mutations (blue). (TIF) [file pgen.1007779.s007.tif]

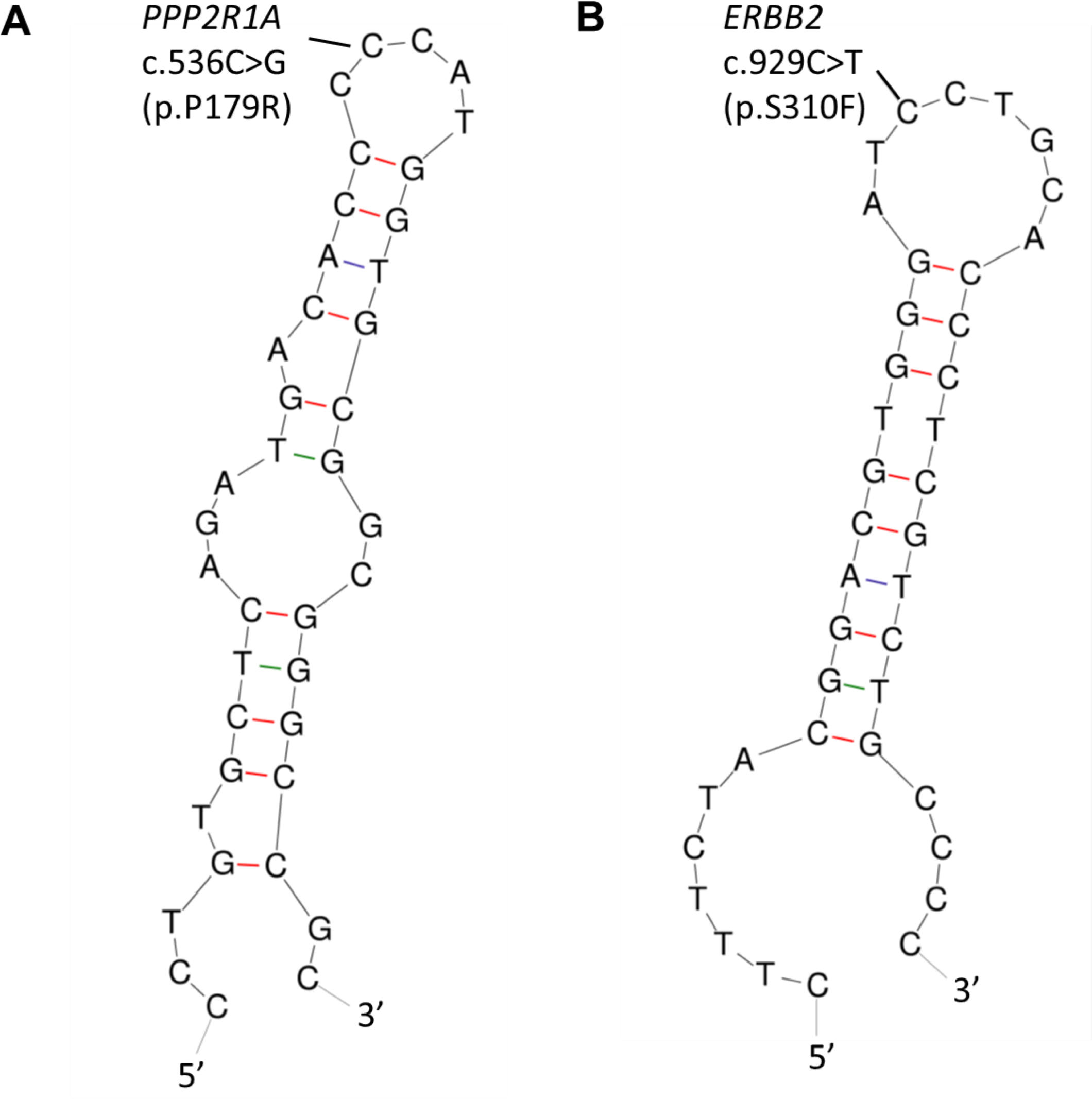

Supplement: S4 Fig — Predicted ssDNA secondary structure shown for (A) PPP2R1A p.P179R and (B) ERBB2 p.S310F mutations. The mutated base is denoted by the mutation label. Predictions were made using the mFold prediction tool [46] with default parameters. (TIF) [file pgen.1007779.s008.tif]

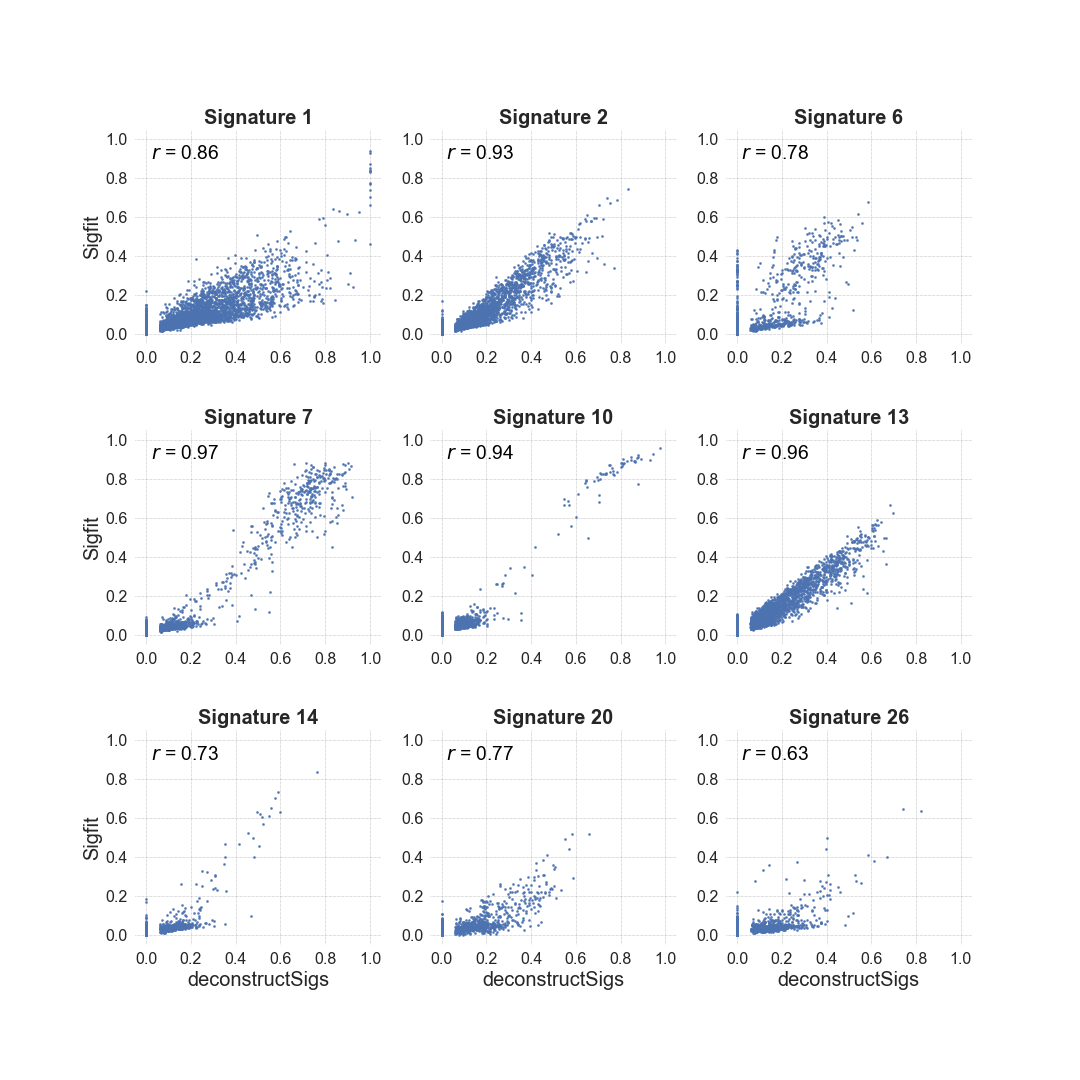

Supplement: S5 Fig — The proportion of mutations attributed to each mutational signature is shown for Sigfit (y-axis) and DeconstructSigs (x-axis), where dots indicate individual samples. The Pearson’s correlation (r) is indicated for each signature on individual plots. (TIF) [file pgen.1007779.s009.tif]
